# The UBE2J2/UBE2K-MARCH5 ubiquitination machinery regulates apoptosis in response to venetoclax in acute myeloid leukemia

**DOI:** 10.1038/s41375-024-02178-x

**Published:** 2024-02-16

**Authors:** Shan Lin, Constanze Schneider, Angela H. Su, Gabriela Alexe, David E. Root, Kimberly Stegmaier

**Affiliations:** 1 Department of Pediatric Oncology, Dana-Farber Cancer Institute, Boston, MA USA; 2 Broad Institute of MIT and Harvard, Cambridge, MA USA; 3 Division of Hematology/Oncology, Boston Children’s Hospital, Boston, MA USA

**Keywords:** Acute myeloid leukaemia, Apoptosis

Evasion of apoptosis is crucial for the growth, survival and chemoresistance of many cancer types, including acute myeloid leukemia (AML); thus, the reactivation of apoptosis can be exploited as a therapeutic approach. Apoptosis induction is mainly controlled by the balance between anti-apoptotic and pro-apoptotic BCL2 family proteins, which determines the oligomerization of effectors BAX and BAK and thus the permeabilization of mitochondrial membranes [1]. Venetoclax, a selective inhibitor antagonizing the anti-apoptotic protein BCL2, has emerged as a promising therapy in AML. Despite high response rates in combination with hypomethylating agents, some patients display upfront resistance, and most patients will ultimately relapse [2–4]. Therefore, identification of synergistic targets for combination therapies with venetoclax is important for improving the clinical application of this drug. Several potential targets have been revealed. For instance, inhibiting BCLXL or MCL1, two anti-apoptotic BCL2 family members, can trigger synergistic anti-tumor activity with venetoclax [2]. We also reported that inhibition of the RING-type ubiquitin E3 ligase MARCH5 can induce BAX/BAK-dependent apoptosis and lead to venetoclax sensitization in AML cells [5].

**Fig. 1:**
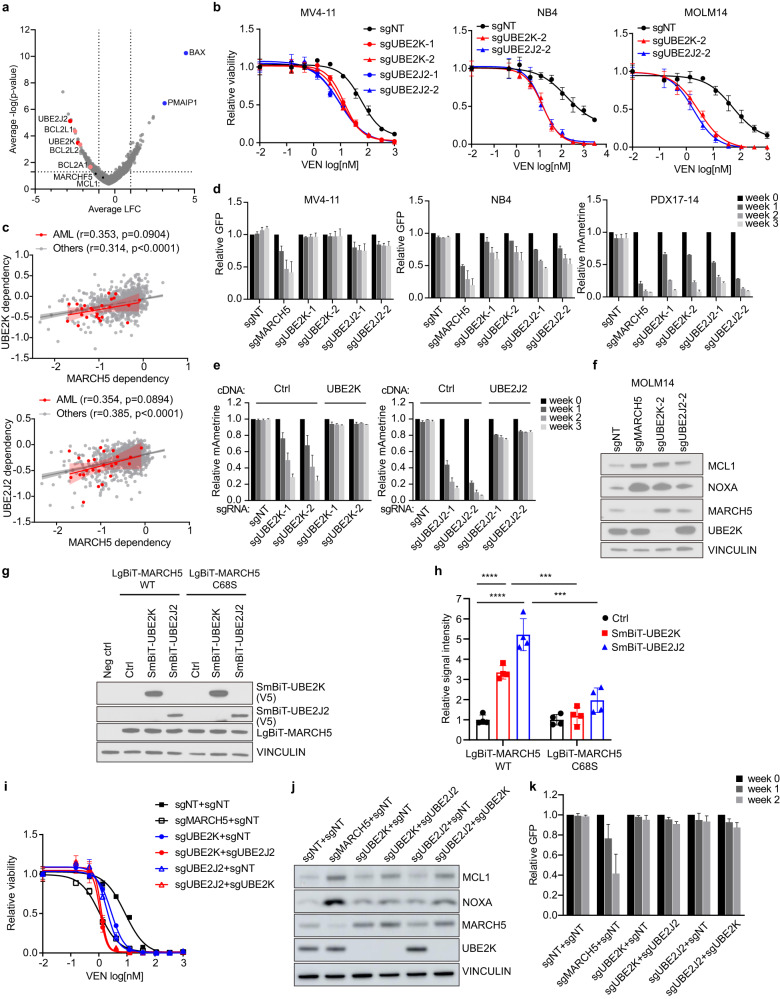
Identification of UBE2J2 and UBE2K as the collaborating E2 proteins of MARCH5. **a** Scatterplot showing the log_2_ fold-change (LFC) of sgRNA abundance and the associated *p*-value in venetoclax-treated cells compared to DMSO control cells. The scores of all sgRNAs targeting the same gene were averaged and shown as one data point. **b** Relative viability of AML cells expressing the indicated sgRNAs after 3-day venetoclax (VEN) treatment. Cell viability was determined by CellTiter-Glo and normalized to the DMSO-treated control. The mean ± SD (*n* = 4) and dose-response curves are plotted. sgNT, a non-targeting sgRNA. **c** Scatterplot showing the Pearson correlations between the dependency scores of *MARCH5* and *UBE2J2* or *UBE2K* across AML cell lines or other cancer cell lines in the DepMap dataset. Each dot represents a cell line. **d** In vitro competitive growth of AML cells co-expressing the indicated sgRNAs and GFP. Relative cell growth is measured by the change in the percentage of GFP+ cells; results represent mean + SD, *n* = 2. **e** Competitive growth of PDX17-14 cultured cells transduced with an empty vector (Ctrl) or the CRISPR-resistant cDNA of *UBE2J2* or *UBE2K* upon the knockout of the corresponding endogenous E2 genes; results represent mean + SD, *n* = 2. **f** Immunoblot analysis of MCL1 and NOXA upon *MARCH5*, *UBE2J2* or *UBE2K* knockout in MOLM14 cells. **g** Immunoblot analysis confirming the expression of LgBiT-tagged MARCH5 proteins and SmBiT-tagged E2 proteins in transduced MV4-11 cells. Non-transduced cells were used as a negative control (neg ctrl). **h** Relative luminescent signals of MV4-11 cells expressing the indicated NanoBiT constructs; results represent mean ± SD, *n* = 4. ***p < 0.001; ****p < 0.0001, unpaired two-sided *t*-test. **i**. Relative cell viability with venetoclax treatment (mean ± SD, *n* = 3) of MV4-11 cells transduced with the indicated sgRNA combinations. Immunoblot analysis to confirm the gene knockout **j** and competition proliferation assays to evaluate the growth effect of E2 depletion as single or double-knockout in MV4-11 (mean + SD, *n* = 2) **k**.

To systematically identify other key genes that can modulate the venetoclax effect, we performed a genome-scale CRISPR-Cas9 screen in the human AML cell line MV4-11. MV4-11 cells were transduced with the Avana genome-scale sgRNA library, which targets each gene with four sgRNAs. Transduced cells were divided into two groups and cultured with the vehicle control DMSO or 10 nM venetoclax for 16 days at which point cell pellets were collected for sequencing analysis (Supplementary Fig. 1a). This concentration of venetoclax resulted in a 30% decrease in cell viability, allowing us to identify gene targets whose loss can either confer resistance or induce sensitization. Significantly enriched or depleted sgRNAs in venetoclax-treated cells compared to DMSO controls were identified. The hypergeometric method was used to assess *p*-values and the log_2_ fold-change (LFC) of the set of sgRNAs targeting the same gene. Consistent with previous reports, loss of the apoptosis effector *BAX* or the pro-apoptotic gene *NOXA* (*PMAIP1*) caused venetoclax resistance, while sgRNAs targeting the anti-apoptotic genes *BCLXL* (*BCL2L1*), *BCL2L2* and *BCL2A1* were significantly more depleted in venetoclax-treated cells (Fig. 1a) [6–8]. *MCL1* and *MARCH5* did not score in this analysis because deletion of these two targets strongly inhibits the growth of MV4-11 cells, causing a strong depletion of their sgRNAs in both treatment and control groups.

We next focused on the E2 ubiquitin-conjugating enzymes UBE2J2 and UBE2K, two highly ranked venetoclax sensitizers that have not been well characterized (Fig. 1a). We validated that depletion of either *UBE2J2* or *UBE2K* increased sensitivity to venetoclax in MV4-11 and two additional AML cell lines (Fig. 1b). Successful gene knockout was confirmed by immunoblot for *UBE2K* and genomic loci sequencing for *UBE2J2* (Supplementary Fig. 1b, c).

Intriguingly, exploiting the Broad Institute’s Cancer Dependency Map (DepMap) dataset (https://depmap.org/), which includes genome-scale CRISPR-Cas9 screens in over 1000 cancer cell lines, revealed that dependency on *UBE2J2* or *UBE2K* significantly correlated with a dependency on *MARCH5* across all cancer cell lines; and similar trends were observed within AML cell lines (Fig. 1c). Since E2 enzymes coordinate with ubiquitin E3 ligases to execute ubiquitination processes, this observation suggests that UBE2J2 and UBE2K can cooperate with MARCH5 to exert its biological function. We validated the dependency association between *MARCH5* and the two E2s using in vitro competition assays. Deletion of either E2 elicited stronger growth inhibitory effects in the AML cells that were more dependent on *MARCH5*, particularly in cultured cells from a CRISPR-competent patient-derived xenograft (PDX) model of AML (PDX17-14, complex karyotype with an MLL-AF10 fusion) [5] (Fig. 1d). Leveraging this model, we performed rescue experiments with CRISPR-resistant cDNAs and confirmed the on-target effects of *UBE2J2* and *UBE2K* sgRNAs (Fig. 1e). Notably, *UBE2J2* and *UBE2K* knockout led to the upregulation of MCL1 and NOXA, two reported substrates of MARCH5, albeit to a lesser extent than with *MARCH5* knockout [9]. Interestingly, MARCH5 protein levels were also increased upon *UBE2K* and *UBE2J2* deletion, suggesting that the E2s may also be involved in MARCH5 self-ubiquitination (Fig. 1f). Next, we utilized the NanoBiT technology [10], a structural complementation reporter system, to detect protein interactions between MARCH5 and the E2 candidates in AML cells. LgBiT and SmBiT, two split subunits of luciferase, were fused with MARCH5 and the E2 proteins, respectively. The luminescent signal was activated upon the co-expression of LgBiT-MARCH5 with either of the SmBiT-tagged E2 proteins but not an empty vector. In contrast, introducing the C68S mutation into LgBiT-MARCH5, which disrupts the RING domain and thus the E2-binding capacity of MARCH5, largely diminished the luminescent signal (Fig. 1g, h), supporting the specificity of our assays. Collectively, our data suggests that MARCH5 and UBE2J2/UBE2K constitute ubiquitination machinery that regulates apoptosis in AML.

Given that *UBE2J2* or *UBE2K* single knockout induced milder downstream effects compared to *MARCH5* deletion, we next assessed the possible redundancy between the two E2s. Indeed, the double knockout (DKO) of *UBE2J2/UBE2K* further enhanced venetoclax sensitivity (Fig. 1i). However, *UBE2J2/UBE2K* DKO was not able to upregulate the downstream substrates or induce the defective growth of AML in the absence of venetoclax to the same degree as MARCH5 depletion (Fig. 1j, k and Supplementary Fig. 1d), indicating that MARCH5 may collaborate with additional E2s to compensate for the loss of UBE2J2 and UBE2K.

**Fig. 2:**
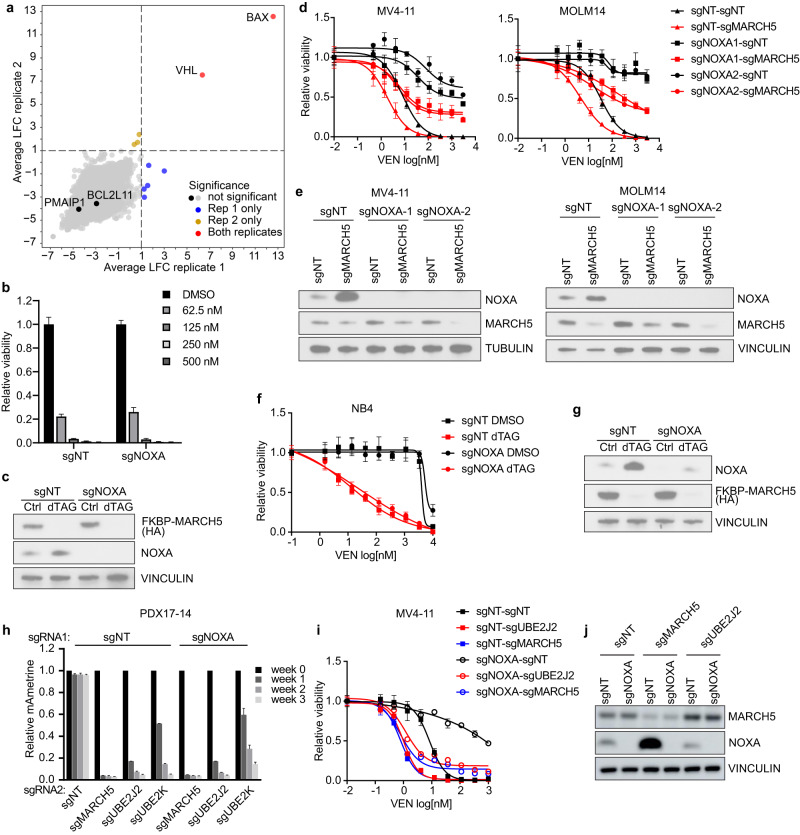
MARCH5 ubiquitination machinery regulates AML apoptosis in a NOXA-independent manner. **a** Scatterplot showing the log2 fold-change (LFC) of sgRNA abundance in dTAG^V^1-treated cells compared to DMSO control cells in duplicates. The scores of all sgRNAs targeting the same gene were averaged and shown as one data point. The significant enriched targets in each replicate (average LFC > 1 and *p*-value < 0.05) are indicated by color codes. **b** Relative viability of control (sgNT) or *NOXA*-deleted (sgNOXA) PDX17-14 dTAG-MARCH5 cells treated with DMSO or dTAG^V^-1 at the indicated concentrations for three days. Cell viability was determined by CellTiter-Glo and normalized to the DMSO-treated cells. The plots represent mean ± SD (*n* = 4). **c** Immunoblot analysis of the cells in **b** treated with DMSO (Ctrl) or 500 nM dTAG^V^-1 (dTAG) for 24 h. **d** Relative viability of control or *NOXA*-knockout AML cells with or without *MARCH5* deletion upon venetoclax (VEN) treatment. The mean ± SD (*n* = 4) and dose-response curves are plotted. **e** Immunoblot analysis of the cells used in **d** to confirm NOXA and MARCH5 depletion. **f** Relative viability of control or *NOXA*-deleted NB4 dTAG-MARCH5 cells treated with venetoclax for 3 days (mean ± SD, *n* = 4). Cells were treated with DMSO or 500 nM dTAG^V^-1 concurrently with venetoclax treatment. **g** Immunoblot analysis of the cells used in **f** treated with DMSO (Ctrl) or 500 nM dTAG^V^-1 (dTAG) for 24 h. **h**. Competition proliferation assays to evaluate the growth effect of MARCH5 or E2 depletion in control and *NOXA*-knockout PDX17-14 cells; results represent mean + SD, *n* = 2. Relative cell viability with venetoclax treatment (mean ± SD, *n* = 3) **i**, and immunoblot analysis **j** of MV4-11 cells transduced with the indicated sgRNA combinations.

As shown by our studies and those of others [5, 9], MARCH5 depletion increased NOXA protein levels, which is an important node in dictating venetoclax response. Several reports indicated that NOXA was a critical downstream target of MARCH5 in controlling cellular responses to various anti-cancer treatments [9, 11–13]. In contrast, we previously showed that MARCH5 can regulate apoptosis independently of NOXA in AML [5]. To reassess the role of NOXA and other pro-apoptotic proteins in MARCH5-mediated apoptosis, we conducted an unbiased CRISPR rescue screen in a MARCH5-dTAG degradation system derived from PDX17-14 cells [5]. MARCH5 degradation and the ensuing cell death can be induced with the molecule dTAG^V^-1 in this model. The MARCH5-dTAG cells were transduced with the Avana sgRNA library in duplicates. Fourteen days post transduction, each replicate was split into two groups and treated with DMSO or dTAG^V^-1. Cell pellets were collected 10 days after treatment, and sequencing analysis was performed to identify the sgRNA targets that were positively selected in the dTAG^V^-1-treated cells compared to the controls. The E3 ligase *VHL* scored strongly in both replicates as an expected quality control (Fig. 2a), since dTAG^V^-1 binds VHL to achieve targeted protein degradation [14]. Thus, deletion of *VHL* will block MARCH5 degradation and cell death. *BAX* was the top rescuing target, emphasizing that apoptosis induction is the main mechanism accounting for the growth inhibition of MARCH5-depleted cells. However, deletion of other pro-apoptotic BCL2 members, including *NOXA* (*PMAIP1*) and *BIM* (*BCL2L11*), did not rescue MARCH5 depletion in this screen, consistent with our previously published data [5] (Fig. 2a). In fact, the PDX17-14 MARCH5-dTAG cells with or without *NOXA* deletion were equally vulnerable to dTAG^V^-1-mediated MARCH5 degradation (Fig. 2b, c).

Our previous study showed that venetoclax resistance induced via *NOXA* knockout can be attenuated by MARCH5 depletion in MV4-11 cells [5]. Here we confirmed the observation in additional AML cell lines with both CRISPR and dTAG approaches (Fig. 2d–g). In accordance, *UBE2J2* and *UBE2K* regulated the apoptosis response in a similar manner as MARCH5. Knockout of *UBE2J2* or *UBE2K* can repress the cell growth of PDX17-14 irrespective of the *NOXA* status (Fig. 2h). Additionally, *UBE2J2* or *UBE2K* deletion increased venetoclax sensitivity even in the *NOXA*-null cells of multiple AML models, including another PDX-derived model cultured briefly in vitro (PDX16-01, with *CALM-AF10* fusion, *NF1*, *PHF6* and *TP53* mutations) (Fig. 2i, j and Supplementary Fig, 2a–d). Even in the AML models where *UBE2J2* and *UBE2K* were weak dependencies by themselves, the mild defective growth cannot be rescued by *NOXA* knockout. Moreover, enhancement of venetoclax response with E2 targeting remained present regardless of *NOXA* expression (Supplementary Fig. 2e, f).

Corroborating a recent report [15], our study highlights that UBE2J2 and UBE2K are two important functional partners of MARCH5 in regulating apoptosis in AML cells and can serve as additional targets for enhancing venetoclax efficacy. However, in contrast to this recent report and consistent with our previously published study [5], unbiased screening and low-throughput target validation further emphasize that the MARCH5 ubiquitination machinery regulates apoptosis in AML cells largely in a NOXA-independent manner in the absence of venetoclax treatment.

As reported previously, knockout of *NOXA* strongly renders resistance to venetoclax in multiple models of AML [6, 7, 15]. We consistently observed increased sensitivity to venetoclax with MARCH5 depletion, even in the context of *NOXA* knockout, although not to the same extent as with *MARCH5* knockout alone. Thus, our data and supplementary data presented in a prior report [15] are consistent with MARCH5 also playing a NOXA-independent role in regulating response to venetoclax treatment. Additional studies are needed to dissect this complex MARCH5/UBE2J2/UBE2K mediated apoptosis regulation in AML.

### Supplementary information

Supplementary Methods

Supplementary Figure 1

Supplementary Figure 2

Supplementary Figure legends
