# Supplementary material for: The UBE2J2/UBE2K-MARCH5 ubiquitination machinery regulates apoptosis in response to venetoclax in acute myeloid leukemia
Source: Leukemia. 2024 Feb 16;38(3):652–6. doi: 10.1038/s41375-024-02178-x (PMC10912020; doi:10.1038/s41375-024-02178-x)
Supplement: Supplementary file 1 — Supplementary Methods [file 41375_2024_2178_MOESM1_ESM.docx]

**Methods**

**Plasmids and reagents**

The Cas9-T2A-mCherry vector and the inducible sgRNA vector co-expressing GFP were obtained from Addgene (#70182 and #70183). The lentiviral sgRNA expression vectors co-expressing a puromycin resistance gene or mAmetrine (pXPR_003, pXPR_052) were provided by the Genetic Perturbation Platform (GPP) at the Broad Institute. sgRNAs were cloned into BsmBI-digested vectors. Double knockout experiments were performed using two sgRNA vectors with different reporters. sgRNA sequences are provided in the following table:

| sgNT-1 | CAGAGCCTTGCGCAATTTTG | sgUBE2K-1 | AGCTGCAATGACTCTCCGCA |
| --- | --- | --- | --- |
| sgNT-2 | CCGCGCATTTCAGAGCACAA | sgUBE2K-2 | GCAATGACAATAATACCGTG |
| sgMARCH5-1 | GTAGATCCTCTGCACCTGCA | sgUBE2J2-1 | ATATGATCACTCCCAACGGG |
| sgNOXA-1 (PMAIP1) | ACGCTCAACCGAGCCCCGCG | sgUBE2J2-2 | GGAGGTTTAAGTGCAACACC |
| sgNOXA-2 (PMAIP1) | GGTACCTGCTGGAGCCCGCG |  |  |

For overexpression, cDNAs of UBE2K, UBE2J2 and for the NanoBiT-tagged proteins were synthesized as gBlocks fragments (Integrated DNA Technologies), and then cloned into the lentiviral expression vectors co-expressing a puromycin resistance gene or GFP (pLX_TRC307 and pLX_TRC312 from GPP) using a Gibson Assembly Cloning Kit (New England Biolabs E5510S).

Venetoclax was acquired from Selleck (S8048) or MedChemExpress (HY-15531), Doxycycline was acquired from Takara Bio (631311) and dTAG^V^-1 compound was kindly provided by Dr. Nathanael Gray.

**Cell culture**

All commercially available cell lines were obtained from ATCC or DSMZ. MV4-11, MOLM14 and NB4 cells were cultured in RPMI supplemented with 10% fetal bovine serum (FBS) and 1% penicillin-streptomycin (PS). HEK293T cells were cultured in DMEM with 10% FBS and 1% PS. During the preparation of this study, the mycoplasma negativity was confirmed using a LookOut Mycoplasma PCR Detection Kit (Sigma Aldrich MP0035), and the identity of all cell lines were validated through short tandem repeat (STR)-profiling by the Molecular Diagnostics Laboratory at the Dana-Farber Cancer Institute (DFCI).

The CRISPR-competent patient-derived xenograft (PDX) model PDX17-14 and MARCH5-dTAG models were established previously^1^. PDX cells were maintained in IMDM containing 20% FBS and 1% PS, and supplemented with 10 ng/mL human SCF, TPO, FLT3L, IL-3 and IL-6 (PeproTech 300-07, 300-18, 300-19, 200-03 and 200-06).

**Lentivirus production and transduction**

Virus was produced using HEK293T cells transfected with lentiviral expression vectors, together with the envelope VSVG and the gag-pol psPAX2 constructs. For transduction, AML cells were mixed with viral supernatant and 4-8 µg/mL polybrene. In some experiments, cells were centrifuged in viral supernatant at 1000 g for 1hr at 33 °C to enhance transduction efficiency.

**CRISPR screens**

Venetoclax modifier screen. MV4-11 Cas9-mCherry positive cells were transduced with the Avana CRISPR knockout library (lentiCRISPRv2) in two biological replicates to achieve 30-40% infection efficiency^2^. Twenty-four hours after transduction, cells were pooled and selected with puromycin for 72 hours. After selection, cells were counted and split in two sets of 50x10^6^ cells per replicate, to maintain a library representation of >500 cells per sgRNA. Treatment with DMSO or 10 nM venetoclax (SelleckChem #S8048) started on day 7 after transduction. Cells were counted, split, and retreated every 4 days; surviving cells were harvested after 16 days. Genomic DNA was extracted from the collected cell pellets using a NucleoSpin Blood L kit (Takara #740954.20). sgRNA sequences were PCR amplified and submitted for standard Illumina sequencing as previously described^3^. In each sample, the read counts of each sgRNA were normalized to the total reads per million. The fold-change of normalized sgRNA counts in venetoclax-treated samples was determined relative to the DMSO-treated samples for each replicate, which were then log2 transformed and averaged across the two replicates (LFC). The p-values indicating the significant enrichment or depletion of each sgRNA were calculated using a probability mass function of hypergeometric distribution based on the sgRNA LFC ranking. The average LFC and p-values of all sgRNAs targeting the same gene were used as gene-level scores; only genes with at least three sgRNAs mapped were reported.

dTAG-MARCH5 rescue screen. This screen was conducted similarly as above. Briefly, PDX17-14 dTAG-MARCH5 cells were transduced with the Avana CRISPR library in duplicates at a ~40% efficiency and selected with puromycin. Cells were treated with DMSO or 500 nM dTAG^V^-1 at day 14 post puromycin selection for 10 days and then collected for sequencing analysis. The data was processed similarly except that gene-level scores were calculated for each replicate individually instead of as the average value of two replicates.

**TIDE (Tracking of Indels by DEcomposition) analysis of sgRNA editing**

To confirm the on-target editing of *UBE2J2*, *UBE2J2* sgRNA-targeted genomic DNA regions were amplified via PCR using the NEBNext High-Fidelity 2x PCR Master Mix (New England Biolabs #M0541) and the following primers: forward 5´-GACCTGAGTCCTGAGAGGGG; reverse 5´- AACTTACCGTGAAGTCCGAC-3´. PCR products were purified using a QIAquick PCR purification kit (QIAGEN #28104) and sent for Sanger sequencing with the reverse primer. The sequencing results were analyzed by the TIDE tool (http://shinyapps.datacurators.nl/tide/)^4^ to infer editing efficiencies. Sequencing results from a non-target (sgNT) sample were used as controls.

**Western blotting**

Cell lysates were resolved in SDS-PAGE, followed by transfer to PVDF membranes and immunoblotting. The primary antibodies used were anti-MARCH5 (Cell Signaling Technology (CST) #19168), anti-UBE2K (CST #8226), anti-VINCULIN (CST #13901), anti-HA (CST #3724), anti-V5 (CST #13202), anti-MCL1 (#94296 and #5453), anti-NOXA (CST #14766) and anti-TUBULIN (MilliporeSigma T0198). The secondary antibodies were HRP-linked goat anti-rabbit IgG and anti-mouse IgG (CST #7074, #7076).

**Cell viability assay**

AML cells were plated in 384-well plates at 1,500-2,000 cells (for MV4-11, NB4 and MOLM14) or 10,000 cells (for PDX17-14) in 50 µL medium per well and mixed with serially-diluted concentrations of venetoclax, or dTAG^V^-1, or 0.1% DMSO as a control. The viability of cells was measured after 3 days incubation using a CellTiter-Glo Luminescent Cell Viability Assay kit (Promega #G7571) following the manufacturer’s protocol. Data were analyzed using GraphPad Prism software.

**Competition growth assay**

Cells were transduced with lentivirus vectors co-expressing a sgRNA and a fluorescent protein, such as GFP or mAmetrine, at an efficiency of approximately 50%, or the transduced cells were mixed with non-transduced cells at approximately a 1:1 ratio. The cell growth was evaluated by the change in the fraction of cells expressing the fluorescent protein, which was monitored by flow cytometry on an LSRFortessa or FACSCelesta flow cytometer (BD Biosciences). The flow cytometry data was analyzed with FloJo software (TreeStar).

**NanoBiT assay**

MV4-11 cells stably expressing the NanoBiT constructs were established via lentiviral transduction. The cells were seeded in 96-well plates at 2X10^5^ in 100 µL medium per well. The Nano-Glo Live Cell Assay reagent (Promega #N2011) was added 3 hours later following the manufacturer’s protocol, and luminescence was measured after 10 minutes incubation.

**Supplemental references**

1. Lin S, Larrue C, Scheidegger NK, Seong BKA, Dharia NV, Kuljanin M*, et al.* An In Vivo CRISPR Screening Platform for Prioritizing Therapeutic Targets in AML. *Cancer Discov* 2022 Feb; **12**(2)**:** 432-449.

2. Doench JG, Fusi N, Sullender M, Hegde M, Vaimberg EW, Donovan KF*, et al.* Optimized sgRNA design to maximize activity and minimize off-target effects of CRISPR-Cas9. *Nat Biotechnol* 2016 Feb; **34**(2)**:** 184-191.

3. Meyers RM, Bryan JG, McFarland JM, Weir BA, Sizemore AE, Xu H*, et al.* Computational correction of copy number effect improves specificity of CRISPR-Cas9 essentiality screens in cancer cells. *Nat Genet* 2017 Dec; **49**(12)**:** 1779-1784.

4. Brinkman EK, Chen T, Amendola M, van Steensel B. Easy quantitative assessment of genome editing by sequence trace decomposition. *Nucleic Acids Res* 2014 Dec 16; **42**(22)**:** e168.
