# Supplementary figures and images for: The UBE2J2/UBE2K-MARCH5 ubiquitination machinery regulates apoptosis in response to venetoclax in acute myeloid leukemia
Source: Leukemia. 2024 Feb 16;38(3):652–6. doi: 10.1038/s41375-024-02178-x (PMC10912020; doi:10.1038/s41375-024-02178-x)

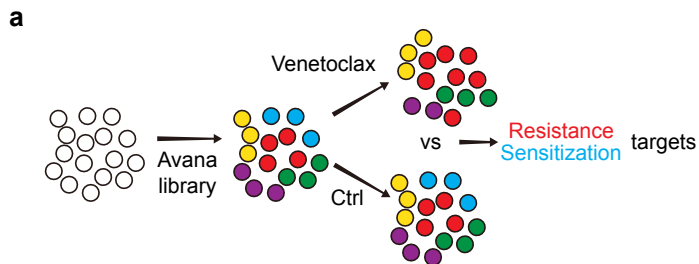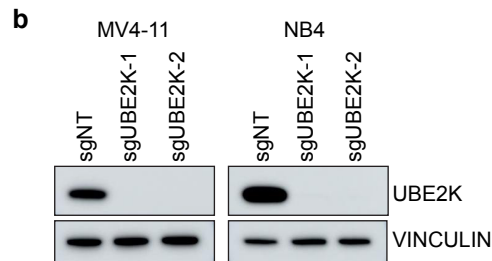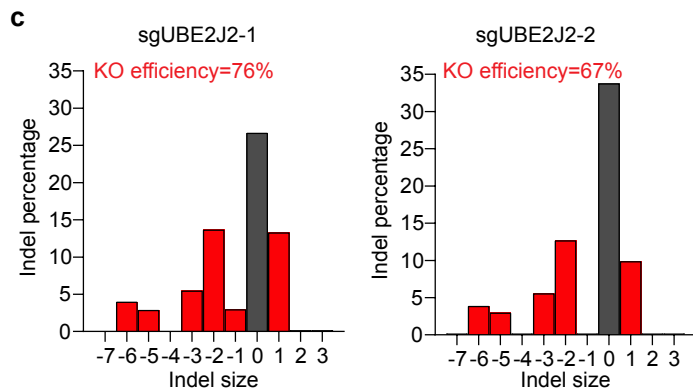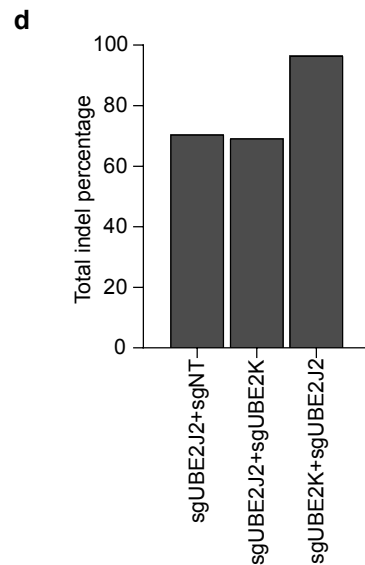

Supplement: Supplementary file 2 — Supplementary Figure 1 [file 41375_2024_2178_MOESM2_ESM.pdf]

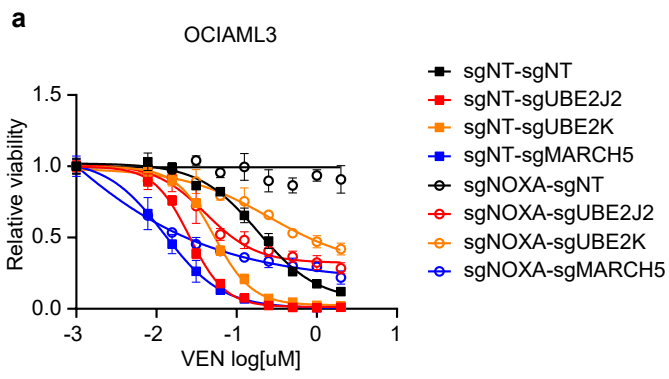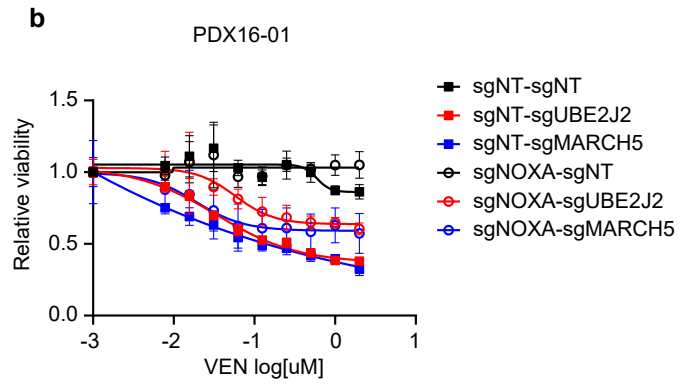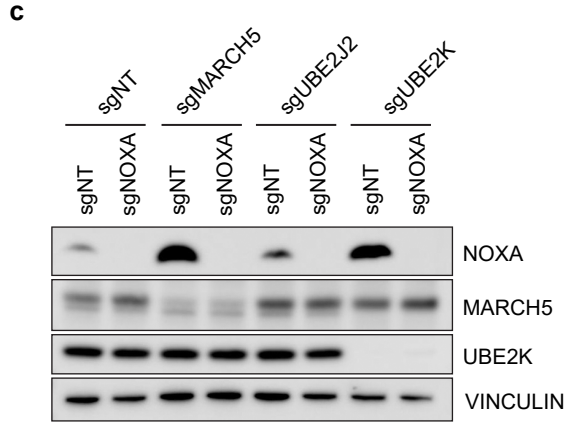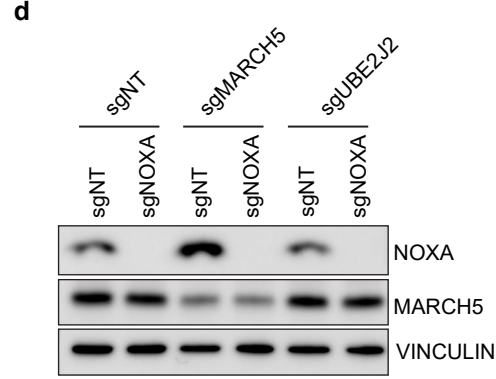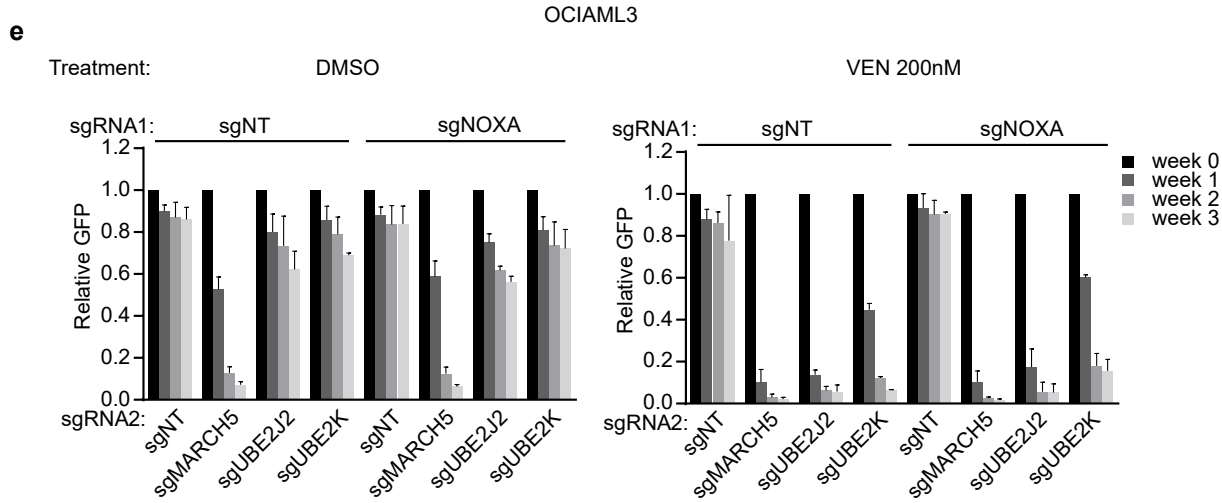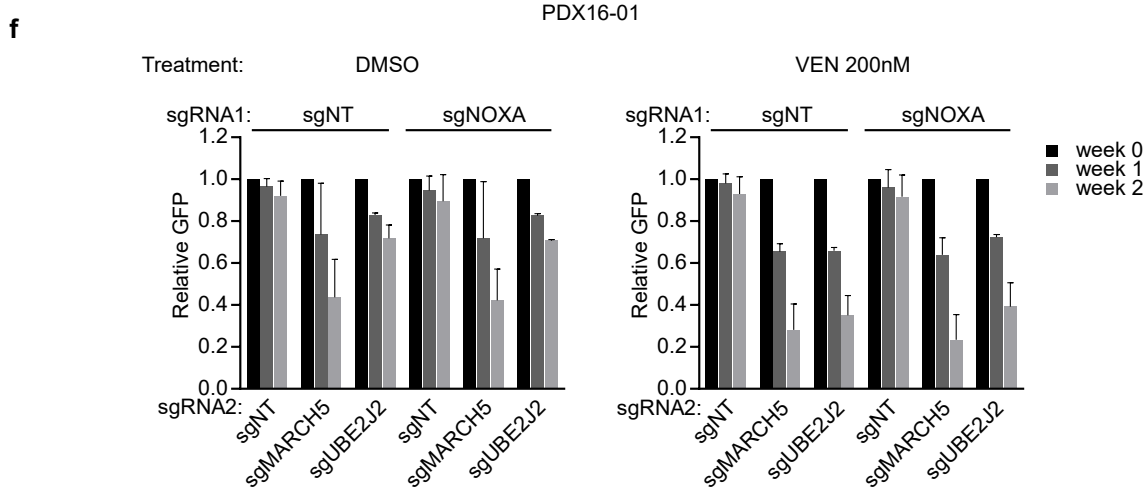

Supplement: Supplementary file 3 — Supplementary Figure 2 [file 41375_2024_2178_MOESM3_ESM.pdf]
