# Supplementary material for: The UBE2J2/UBE2K-MARCH5 ubiquitination machinery regulates apoptosis in response to venetoclax in acute myeloid leukemia
Source: Leukemia. 2024 Feb 16;38(3):652–6. doi: 10.1038/s41375-024-02178-x (PMC10912020; doi:10.1038/s41375-024-02178-x)
Supplement: Supplementary file 4 — Supplementary Figure legends [file 41375_2024_2178_MOESM4_ESM.docx]

**Supplementary Figure 1. Validation of UBE2J2 and UBE2K knockout.**

**a.** Schematic for identifying the regulators of venetoclax response in AML by CRISPR screen. **b.** Immunoblot analysis to confirm the depletion of UBE2K in MV4-11 and NB4 cells. **c.** Quantitative assessment of the knockout (KO) efficiency of *UBE2J2* in MV4-11 cells transduced with the indicated sgRNAs. Sanger sequencing with tracking of indels by computational decomposition (TIDE) analysis to show the percentage of *UBE2J2* loci harboring indels with the indicated sizes (red bars) and unedited allele (black bars). Sequences from sgNT-expressing cells were used as references. The KO efficiency was represented by the total percentage of *UBE2J2* loci with an indel. **d.** Percentage of edited *UBE2J2* loci in MV4-11 cells transduced with the indicated sgRNA combinations.

**Supplementary Figure 2. Depletion of UBE2J2 or UBE2K increases the venetoclax sensitivity of *NOXA*-null AML cells.**

**a-b.** Relative cell viability with venetoclax treatment (mean ± SD, n=3) of OCIAML3 (**a**) and PDX16-01 (**b**) cells transduced with the indicated sgRNA combinations. **c-d.** Immunoblot analysis to confirm the gene knockout in cells used in **a** and **b**, respectively. **e-f.** Competition proliferation assays to evaluate the growth effect of MARCH5 or E2 depletion in control and *NOXA*-knockout OCIAML3 (**e**) and PDX16-01 (**f**) cells, treated with DMSO or 200 nM venetoclax; results represent mean + SD, n=2.
